# 

**Published:** 1896-08

**Authors:** 


### CONTENTS.

ORIGINAL ARTICLES:
Some Remarks Bearing upon Tuberculosis. By COOPER CURTICE, M.D., D.V.S. 577
Pathogenesis of Disease. By W. L. RHOADS, D.V.S. . . . . . . . 583
Address of President Stewart before the Missouri Valley Veterinary Associetion . 587
Preventive Inoculation Against Pleuro-pneumonia. By E. BIART, V.S. . . 590
Results of Recent Remarkable Experiments with Young Dogs . . . . . 592
The Lively Flea: A Wonderfully Constructed and Pugnacious Little Pest . . 596
The Dead Horse: How Its Carcass is Utilized in the Arts . . . . . . 597
ABSTRACTS FROM FOREIGN JOURNALS:
Is Mallein a Specific as a Diagnostic Agent?—Contagious Diseases Transferred to Mankind—Aphthous Fever in a Fœtus—Idiosyncrasy against Morphine—Prolapsus Vaginæ in a Filly—Stone in the Bladder of a She-ass—Fistula of the Matrix—On the Use of Turf as Bedding—Two Cases of Rupture of the Uterus —Variability in the Length of Tail in the Dog—Palpation of the Lymphatic Ganglions Accessible to the Touch as a Clinical Diagnostic of Tuberculosis—Melanosis of the Subglossian Ganglions Resembling a Gland in Glanders 599–611
REPORTS OF CASES:
Excision of a Mammary Gland. By W. J. MARTIN, V.S. . . . . . . 612
Translations and Suggestions. By G. LEO HAGENBURGER, M.S., D.V.S. . . 613
CORRESPONDENCE:
Tale of an Unsound Horse . . . . . . . . . . . . . 615
"Query."—What we Want to Know . . . . . . . . . . 616
EDITORIAL:
Buffalo—Our Added Years—Correction—Significant Progress—What Will Our Answer Be?—Our Colleges . . . . . . . . . . 618–621
NECROLOGY . . . . . . . . . . . . . . . . . 622
REVIEWS . . . . . . . . . . . . . . . . . . 625
CONTROL WORK:
New York—Pennsylvania—Louisiana—Maine—Massachusetts—Michigan—Illinois —Kansas—Missouri—National—Great Britain . . . . . . 627–630
AMONG THE COLLEGES:
Harvard—New York State—United States—American—Kansas City . . 631–634
SOCIETY PROCEEDINGS:
Notice to the Members ol the U. S. V. M. A.—Instructions Relative to Railroad Fares—Semi-Annual Meeting of the Illinois State Veterinary Medical Association—Virginia State Veterinary Medical Association—Association of Veterinary Faculties of North America—Niagara and Orleans County Veterinary Association—New York State Veterinary Medical Association . . . . 635–642
GLEANINGS—PERSONAL—DRIFTWOOD—NEW INVENTIONS . . . . 642–648

